# Supplementary material for: Automatic classification of medical image modality and anatomical location using convolutional neural network
Source: PLoS One. 2021 Jun 11;16(6):e0253205. doi: 10.1371/journal.pone.0253205 (PMC8195382; doi:10.1371/journal.pone.0253205)
Supplement: Attachment — Submitted filename: Response to Reviewers.docx [file pone.0253205.s001.docx]

# Rebuttal Letter

Dr. Emily Chenette

Editor-in-Chief

PLOS ONE

May 20, 2021

Dear Dr.Chenette:

Thank you for inviting us to submit a revised draft of our manuscript which entitled, "**Automatic Classification of Medical Image Modality and Anatomical Location Using Convolutional Neural Network**" to PLOS ONE. We also appreciate the time and effort that you and each of the reviewers have dedicated to providing insightful feedback on ways to strengthen our paper. Thus, it is with great pleasure that we resubmit our article for further consideration. We have incorporated changes that reflect the detailed suggestions you have graciously provided. We also hope that our edits and the responses we provide below satisfactorily address all the issues and concerns you and the reviewers have noted.

To facilitate your review of our revisions, the following is a point-by-point response to the questions and comments delivered in your letter dated May 5, 2021.

**EDITOR SUGGESTIONS:**

- Please ensure that your manuscript meets PLOS ONE's style requirements, including those for file naming.

**RESPONSE:** Thank you for your comments. We have addressed this issue by modifying the manuscript according to PLOS ONE’s style.

1. [First reviewer comment]

- Discuss the main motivations of the current work.

**RESPONSE:** Thank you for your comment. We have discussed our motivation in the introduction: The goal of this study is to see if CNN can accurately discriminate medical images from their imaging modalities and anatomical location after they have been converted into other image format, namely JPEG format.

- List out the main contributions of the current work.

**RESPONSE:** Thank you for your comment. We have discussed the main contribution of our work in the abstract section and in the conclusion section by saying: Our study has shown that using CNN for medical image classification is a promising methodology and could work on non-DICOM images, which could potentially save image processing time and storage space.

Also, in our discussion section, we have stated that “despite all previous works, to our knowledge, there were no previous studies that used CNN to classify medical images by both image modality and anatomical location, which is most practical in both clinical and research setting”, which is another main contribution of our work.

- Summarize the drawbacks of existing works in the form of a table.4. Some of the recent works such as the following can be discussed in the paper: "Image-Based malware classification using ensemble of CNN architectures (IMCEC), Deep learning and medical image processing for coronavirus (COVID-19) pandemic: A survey, Hand gesture classification using a novel CNN-crow search algorithm".

**RESPONSE:** This is an important comment and thank you for the reference you have provided. We had a cited this article “Deep learning and medical image processing for coronavirus (COVID-19) pandemic: A survey” in our work. We have also made a new summary table (Table 7) of the references mentioned in our discussion section that are related to medical image classification as you suggested. In our discussion section, we have also discussed regarding the drawbacks of these previous work that we have mentioned.

- Compare the current work with recent state-of-the-art.

**RESPONSE:** Thank you for your comment. Our discussion section had compared the current work with the previous studies that focused on medical image classification on either anatomy and/or image modality. We have also made a summary table (Table 7) of these references in this revision.

- Discuss the limitations of the current work.

**RESPONSE:** Thank you for your comment. We have mentioned several limitations regarding our work in the discussion section. Firstly, we only trained and tested on JPEG images for our CNN architecture. Secondly, we did not perform any image pre-processing for this study and only used the preset window level for our model. Thirdly, we did not choose all the sequences in the two MRI classes. Fourthly, a limited number of test set examples were included in this study. Fifthly, only 4 categories out of two image modalities and three anatomical locations were selected in this study. Please see the discussion section for a more detailed description of our work’s limitation.

2. [Second reviewer comment]

- Please Check the format of the paper

**RESPONSE:** Thank you for this suggestion. We have modified the format of this paper according to PLOS ONE’s style.

- Update the paper lot of Typo's in the whole paper.

**RESPONSE:** Thank you for this suggestion. We have revised our work and corrected the typos that we have found during this proofread.

- Please cite:
  1. Krishna Kagita, M. and M. Varalakshmi, 2020. A detailed study of security and privacy of Internet of Things (IoT). International Journal of Computer Science and Network, 9 (3): 109–113.
  2. Krishna Kagita, M. (2019). Security and Privacy Issues for Business Intelligence in a loT. In Proceedings of 12th International Conference on Global Security, Safety and Sustainability, ICGS3 2019 [8688023]
  3. Navod Neranjan Thilakarathne, Mohan Krishna Kagita, Dr. Thippa Reddy Gadekallu. (2020). The Role of the Internet of Things in Health Care: A Systematic and Comprehensive Study. International Journal of Engineering and Management Research, 10(4), 145-159.

  Visit this profile and cite related papers: <https://scholar.google.com/citations?user=dz4QKZIAAAAJ&hl=en>

**RESPONSE:** Thank you for your suggestion. We have read all three papers that you suggested. However, as these 3 papers were mainly regarding Internet of Things (IoT), which had little relevance to our work, which is about using CNN for medical image classification on both image modality and anatomical location, therefore, we did not cite these papers in this revised version.

**CONCLUDING REMARKS**:

Again, thank you for giving us the opportunity to strengthen our manuscript with your valuable comments and queries. We have worked hard to incorporate your feedback and hope that these revisions persuade you to accept our submission.

Sincerely,

**Hung-Wen Chiu**

Graduate Institute of Biomedical Informatics, Taipei Medical University, Taipei, Taiwan

Address: No. 250 Wu-Xing Street, Taipei, Taiwan (ROC)

E-mail address: hwchiu@tmu.edu.tw
